# The objects of visuospatial short-term memory: Perceptual organization and change detection

**DOI:** 10.1080/17470218.2015.1083595

**Published:** 2015-10-21

**Authors:** Atanaska Nikolova, Bill Macken

**Affiliations:** ^a^ School of Psychology, Cardiff University, Cardiff, UK

**Keywords:** Change detection, Visuospatial short-term memory (VSTM), Cueing, Perceptual organization

## Abstract

We used a colour change-detection paradigm where participants were required to remember colours of six equally spaced circles. Items were superimposed on a background so as to perceptually group them within (a) an intact ring-shaped object, (b) a physically segmented but perceptually completed ring-shaped object, or (c) a corresponding background segmented into three arc-shaped objects. A nonpredictive cue at the location of one of the circles was followed by the memory items, which in turn were followed by a test display containing a probe indicating the circle to be judged *same/different*. Reaction times for correct responses revealed a same-object advantage; correct responses were faster to probes on the same object as the cue than to equidistant probes on a segmented object. This same-object advantage was identical for physically and perceptually completed objects, but was only evident in reaction times, and not in accuracy measures. Not only, therefore, is it important to consider object-level perceptual organization of stimulus elements when assessing the influence of a range of factors (e.g., number and complexity of elements) in visuospatial short-term memory, but a more detailed picture of the structure of information in memory may be revealed by measuring speed as well as accuracy.

Visuospatial short-term memory (VSTM) is typically construed as a set of basic processing mechanisms for the maintenance and manipulation of visuospatial information that underpins a range of cognitive functions, such as orientation and navigation in the environment (Baumann, Skilleter, & Mattingley, 2011) and mental imagery (Prime & Jolicoeur, 2010). Generally, VSTM is associated with maintaining and processing visual and spatial information when that information is no longer present in the immediate surroundings, so it serves a critical function in the construction and maintenance of an integrated representation of the visual world (Prime, Vesia, & Crawford, 2011).

The properties of this system, especially with respect to its capacity, are commonly investigated using the change detection paradigm, which involves brief presentation of a to-be-remembered display, followed by a retention period, followed by a probe display. The probe display can either be identical to or different from the original or some aspect of it. For example, an element may have changed with respect to its location, or a feature may have changed colour or shape, and participants are required to make a *same/different* judgement (e.g., Rouder, Morey, Morey, & Cowan, 2011). Based on such research, debate has centred on issues not only of what the actual capacity limitations of VSTM might be, but also on whether that capacity is better construed as reflecting a discrete slots model, where capacity is limited to about four items (Luck & Vogel, 1997; Zhang & Luck, 2008), or a flexible resource allocation, allowing a trade-off between quantity and quality of items maintained (Bays & Husain, 2008; Huang, 2010). A critical precursor to such issues, however, involves consideration of what are the actual units to which that quantification of capacity is addressed. Therefore, an even more fundamental issue in studying VSTM than that of its capacity is the question of what the functional units are—for example, whether they should be thought of items, or as features, or as some other dimension of the to-be-remembered information (e.g., colour, orientation or size; Hardman & Cowan, 2015).

Importantly for this issue, information in VSTM is often automatically encoded configurally with respect to relational properties of the nominal elements (Boduroglu & Shah, 2014; Brady & Tenenbaum, 2013; Katshu & d'Avossa, 2014). In other words, the operational “items” are rarely, if ever, remembered as independent units of information, and higher order representations are automatically formed, even if irrelevant for the task. Therefore, the question of the relevant units of VSTM has to be addressed not only in a subordinate direction—from items to features—but also in a superordinate one, from items to objects.

The obligatory nature of perceptual organization (Wagemans et al., 2012) and the tendency for the visual system to impose such organization even on impoverished visual scenes (Johansson, 1973; Katz, 1950) means that manipulations of, for example, item complexity or array size may lead to unintended and unacknowledged effects on the emerging perceptual organization of the display. This issue is rarely controlled for in studies of change detection (Orhan & Jacobs, 2014) even though it can have major effects on task performance. For example, research on visual selection has shown that cueing (by introducing a transient peripheral visual event) a location or feature that is part of an object leads to enhanced detection and discrimination of targets in uncued locations within the same object, compared to equidistant targets outside the object: a phenomenon termed object-based attention (Chen, 2012). In addition, this object-based advantage is also evident when the object is perceptually completed by occlusion or illusory contours (Moore & Fulton, 2005; Moore, Yantis, & Vaughan, 1998; Pratt & Sekuler, 2001) so that even physically discontinuous features may combine to operate as integrated objects of selection. These studies demonstrate the compelling influence on performance of the perceptual organization of the visual scene and suggest that perceptually completed objects—whether or not they are physically continuous—form a key functional unit of visual analysis. In turn, this points to the potential influence of the object-level organization of display items in change detection, since visual selection mechanisms and VSTM share common neural networks and constraints (Jonides, Lacey, & Nee, 2005; Postle, 2006).

While it may be that the influence of any such effects would be “washed out” over trials in which the particular locations of the target elements changed randomly from trial to trial, key manipulations designed to probe the precise locus of limitations in performance may introduce systematic effects that cloud interpretation to the extent that they influence performance via processes associated with object formation, rather than by increasing or decreasing some other ostensible factor, such as set size or complexity. This raises the importance of considering the role of perceptual organization and also ensuring that factors that may affect it are held as constant as possible between conditions that involve any spatial rearrangement of the target stimuli themselves, or indeed any changes in any task-irrelevant elements surrounding the stimuli. This represents a real challenge for research on VSTM given the difficulty in defining in advance what items will or will not cohere into single object-level representations. This is especially so since the definition of a perceptual object is not a straightforward one, and object formation can depend on a variety of factors internal and external to any given display and task, including top-down influences, such as previous experience and expectations (Feldman, 1999, 2003; Macken, Taylor, & Jones, 2015; Scholl, 2001).

Indeed, there is evidence to suggest a role for object-level perceptual organization in VSTM similar to that found for reaction times in visual selection tasks (Woodman, Vecera, & Luck, 2003). Woodman et al. (2003) cued one of four locations on the screen followed by a to-be-remembered display consisting of either four squares, each placed at the corners of an imaginary square centred at the midpoint of the screen (*set size 4* condition), or squares arranged in a similar fashion, but with an additional square between either the two horizontal or the two vertical pairs of squares (*set size 6* condition). The latter resulted in changing the perceptual organization of the display into two horizontal or two vertical perceptual objects. After a retention interval, participants had to decide whether one of the squares (the probe) had changed colour compared to the encoding display. Change detection was superior when the probe matched the cued location, compared to probes at uncued locations (cueing effect). However, while in the set size 4 condition there was no accuracy difference between the two probes equidistant from the cue, in the set size 6 condition performance was superior for probes perceptually grouped with the cued location (vertically or horizontally), compared to probes at an equidistant location not grouped with the cue—that is, probes corresponding to squares within the different (uncued) perceptual object (see Woodman et al., 2003). Therefore, perceptual grouping based on Gestalt cues of proximity led to an object-based effect in VSTM in an analogous way to that found in visual selection studies (Chen, 2012).

These results suggest a role for perceptual grouping in the accuracy with which information may be stored or retrieved from VSTM. However, because the manipulation of grouping involved increasing the number and arrangement of items within the display, differences in performance may be an outcome of these changes, rather than being a direct consequence of perceptual object formation per se. Ideally, therefore, the role of object formation needs to be examined in a setting where comparison amongst different object-level conditions can be made without potential confounds from number or spatial arrangement of stimulus elements.

Another issue that we address here relates to precisely how the information represented in VSTM is measured, since while cueing paradigms in the visual selection literature typically measure reaction time, VSTM tasks more commonly measure accuracy of same/different judgements. Such responses to suprathreshold changes in, for example, colour, shape, or location may lack the sensitivity to detect other aspects of the VSTM representations and their retrieval (e.g., Bays & Husain, 2008). So, while a given condition may sustain a particular level of change detection accuracy, measuring the time taken to retrieve the relevant information and make the appropriate decision may reveal aspects of the underlying structure not evident within the accuracy measure. For example, there is often a dissociation between accuracy and reaction time as a measure of visual signal detection, and cueing effects may be more robustly demonstrated in reaction time than in accuracy measures (Smith & Ratcliff, 2009). It may also be the case that measuring speed of processing can reveal variations between conditions when overall accuracy in a task is not expected to show enough variation, for example as a result of ceiling effects (Olson & Marshuetz, 2005). Since our concern here is with potentially subtle effects—that is, the role of perceptual object formation when the spatial relationship between cue and targets remains constant—it may be that a continuous measure such as processing speed will reveal variability undetected in the accuracy of categorical same/different judgements.

To this end, the current study examined the influence of perceptual object formation in VSTM, in a cueing colour change-detection paradigm, while keeping the amount and complexity of visual information in the display as constant as possible across different object-level organizations. Importantly in this respect, we contrasted a stimulus arrangement that should lead to object formation via perceptual completion of a physically occluded object with visually similar conditions in which no such completion should take place, but without the addition or deletion of major stimulus elements (see Figure 1). Participants were required to remember the colours of six briefly presented circles, which were equally spaced and arranged around a central fixation. Their presentation was preceded by a brief transient cue at the location of one of the to-be-remembered circles. After a retention interval, the target circles were presented again with one of them (the probe) surrounded by a bold black outline. Participants had to judge whether or not this probed circle was the same colour as in the original display, and measures of both accuracy and reaction time were taken. The target circles, while always occupying the same positions in the display, appeared within different object-formation conditions as follows: *intact object* condition (Figure 1a), where the target circles appeared within a visibly complete ring superposed over another task-irrelevant object formed of three radiating cones; c*ompleted object* condition (Figure 1b), within which the ring was occluded by the superposition of the second object in such a way as to lead to perceptual completion of the ring (e.g., Moore et al., 1998); and s*egmented object* condition (Figure 1c) in which the second object was still present, and the ring was divided physically and perceptually into separate objects corresponding to the segmented arcs of the ring.

**Figure 1:**
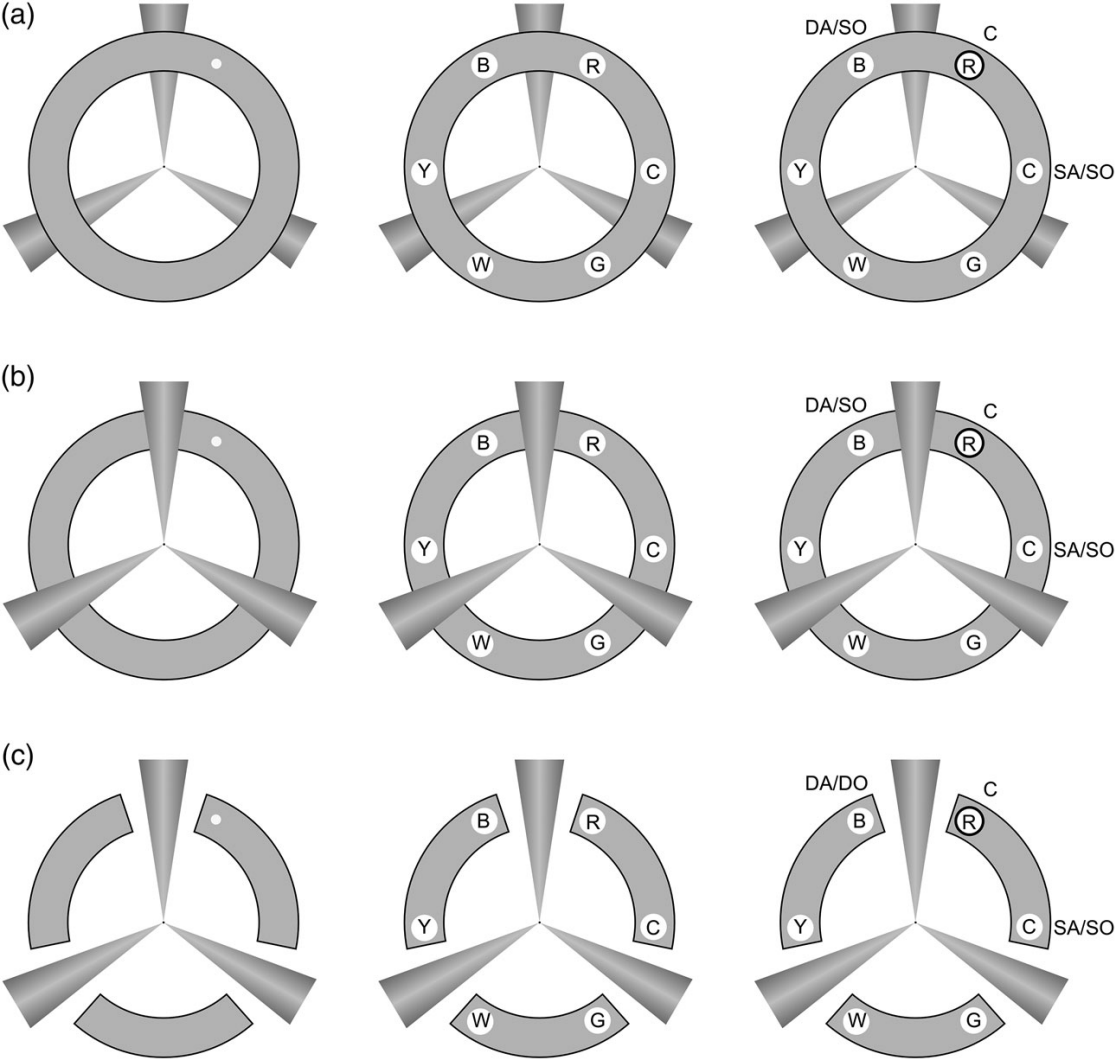
Illustration of the three visual contexts: (a) Intact, (b) Completed, (c) Segmented. The left side of each panel illustrates a possible cue location, the middle illustrates a sample memory array, and the rightmost side depicts a probe at the cued location. The three possible memory probes (relative to the cued location and the separator cone shapes) are labelled as follows: C = cued; SA/SO = same arc/ same object; DA/SO = different arc/ same object; DA/DO = different arc/ different object. Letters inside the target circles indicate the respective colours of the items: R = red, C = cyan, G = green, W = white, B = blue.

Comparison between reaction times and accuracy for the pairs of probe locations lying immediately adjacent to and on either side of the cued location allows us to assess the influence of object-level structure on the representation of information in VSTM. For consistency and clarity across conditions, we refer to the adjacent location beyond the relevant cone of the second object as the *different arc* location, and the other as the *same arc* location, even though in the intact object condition, the ring is physically unsegmented into arcs. If object formation itself constrains the representation of information in VSTM, then this should be revealed by equivalent performance for same and different arc probes in intact and completed conditions, and with superior performance in the segmented object condition for same compared to different arc locations. Furthermore, analysis of the influence of these object-level factors on performance as measured by both accuracy and reaction time should reveal the extent to which each measure is more or less sensitive to the content and structure of information in VSTM.

## EXPERIMENTAL STUDY

### Method

#### Participants

Twenty-eight undergraduate and postgraduate students (4 males), mean age 22.31 years (*SD* = 2.94) were recruited using the Cardiff University, School of Psychology Experiment Management System. The sample had normal or corrected-to-normal vision and normal colour vision. Participants were paid £5 for participation.

#### Stimuli and apparatus

Unless stated otherwise, the size of the stimuli is reported in degrees of visual angle calculated on the basis of a 70-cm viewing distance. Each target circle was 1.0° in diameter, centred at 4.7° from fixation. The six to-be-remembered items were equally spaced, with an angular deviation of 60° relative to the central fixation point. The cue was a small filled circle, 0.52° in diameter, centred on the same axis as the target circles.

All stimuli were presented on a grey background (RGB: 212, 201, 200). The colours of the to-be-remembered items were chosen randomly without replacement from the following set, with the corresponding RGB coordinates in parentheses: brown (205, 133, 63), red (255, 0, 0), yellow (255, 255, 0), green (0, 255, 0), blue (0, 0, 255), cyan (0, 255, 255), and white (255, 255, 255). The cue was coloured in “blanched almond” white (255, 235, 205).

Targets were centred within a ring of 1.95° width and blue-grey colouring (RGB: 200, 200, 200). For the segmented condition, the ring was intersected by three gaps between each pair of memory items. Cone-shaped objects of 7.37° length passed through the middle of each gap, with the thin-end points linked at fixation (Figure 1c). To give the cones the illusion of solid three-dimensionality, their colouring was graded from RGB: 160, 160, 160 on the edges, increasing in steps of 2 units to RGB: 180, 180, 180 at the centre. For the intact condition, the ring appeared superposed on the cones. For the completed condition, the cones occluded the ring. A bold black outline of 0.21° thickness surrounded the probe circle. The whole display (ring and cone shapes) subtended a total of 14.69° × 14.69° centred at fixation.

The experiment was conducted using a Windows XP operating system on a 17″ monitor with 1280 × 1024-pixel resolution and 32-bit colour quality with a 60-Hz refresh rate. A standard keyboard was used to record input. Visual Basic 6.0 was used to program and run the task.

#### Design and procedure

**Figure 2:**
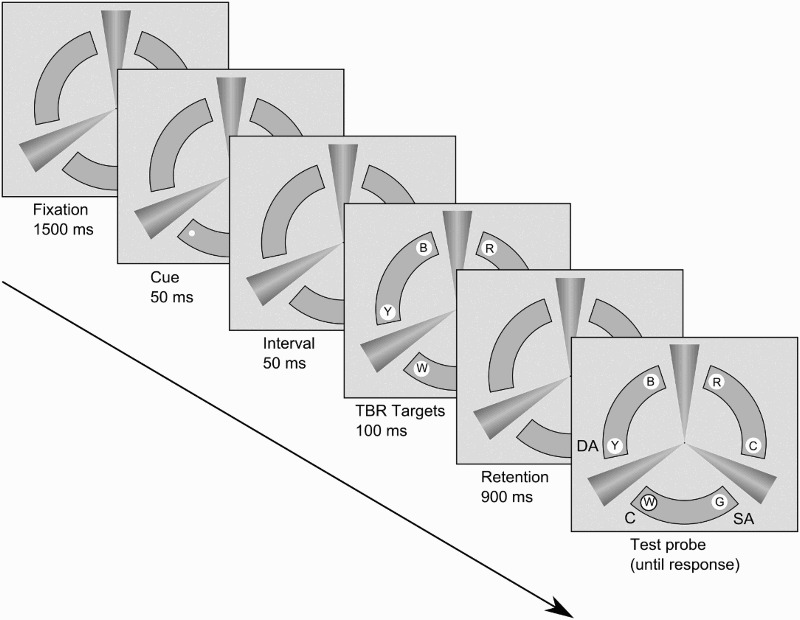
Procedure illustration (Segmented condition). The labels on the far right panel illustrate the three critical probe locations used for the analysis. C: cued; SA: same arc; DA: different arc. In this example memory for the different arc target is probed, which requires a “same” response. TBR = to-be-remembered. Letters inside the target circles indicate the respective colours of the items: R = red, C = cyan, G = green, W = white, B = blue.

A 3 (probe location: cued, same arc, different arc) × 3 (object formation: intact, completed, segmented) repeated measures design was used. The cued probe coincided with the location of the cue (Figure 2, item labelled C). For the purpose of comparison between conditions, the location of the memory items relative to the interpolated cones was used as a landmark to label the two types of uncued probes. Same arc probes were located within the same uninterrupted arc as the cue (Figure 2, item labelled SA), while the different arc probes appeared on the other side of the separating cone (Figure 2, item labelled DA). Thus, in the segmented condition different arc probes were not located on the same intact object as the cue, while in the intact and completed conditions they were on the same perceptual object as the cued location (see Figure 1).

Participants were tested one at a time in semiclosed booths. Each participant underwent a brief practice session with 15 randomized trials, five from each object formation condition. The stimulus without target circles was initially presented for 1500 ms, after which the cue was presented for 50 ms. Participants were instructed that the cue was not informative of the probe's location and should be ignored. Fifty ms after cue offset, the six coloured circles were presented for 100 ms. Following a 900-ms retention interval, the six circles were displayed again with one of them (the probe) surrounded by a bold black outline, indicating that a decision needed to be made about whether it had changed colour from its initial presentation (Figure 2). Participants responded on a standard keyboard by pressing the “<” key for “same” and the “>” key for “different” judgements. These keys were labelled “S” and “D”, respectively.

There were 144 trials for each of the three object formation conditions (432 trials in total). Within each of these, the cue and test probe appeared at random, but with equal probability on each of the six possible locations. Therefore, by the end of the 144 trials, 24 responses were made for each cue–probe relationship. Half of the trials involved a change in probe colour from initial to subsequent presentation, while half were no-change trials. Also, on half of the 144 trials, the location of the dividing cones (and gaps between segments) was randomly rotated by 40° to make sure all possible pairings of targets were used.

The 1480-ms intertrial interval was filled with a dynamic visual noise mask. It consisted of rapidly alternating images of randomly generated black and white pixels and a negative image of the same stimulus configuration as the one in the immediately preceding trial in order to minimize afterimage effects.

There were three blocks, the order of which was counterbalanced between participants, with self-timed breaks in-between. Each block contained a single type of object formation condition. Accuracy (*d*′) and reaction times (ms) were recorded for the three critical locations. The procedure lasted about 45 min.

### Results

The data from two participants were excluded from the analysis. One had a consistently low performance around 50% correct, and for the other a programming error occurred, and more than half of the data were not recorded. As a result, the analysis included the data from 26 participants. A separate 3 × 3 repeated measures analysis of variance (ANOVA) was conducted on accuracy and reaction time for correct responses from the three locations of interest. No responses were trimmed due to prolonged reaction times (the adopted threshold for discarding a trial was 3000 ms). Whenever the assumption of sphericity was violated, Greenhouse–Geisser correction is reported. Bonferroni corrections were applied to all follow-up pairwise comparisons of main effects. Change detection accuracy was measured by transforming the proportion of hits (i.e., when a changed probe was correctly identified as *different*) and false alarms (when the probe colour was unchanged, but the response was *different*) into *z* scores to calculate *d*′ (Macmillan & Creelman, 2004).

**Figure 3:**
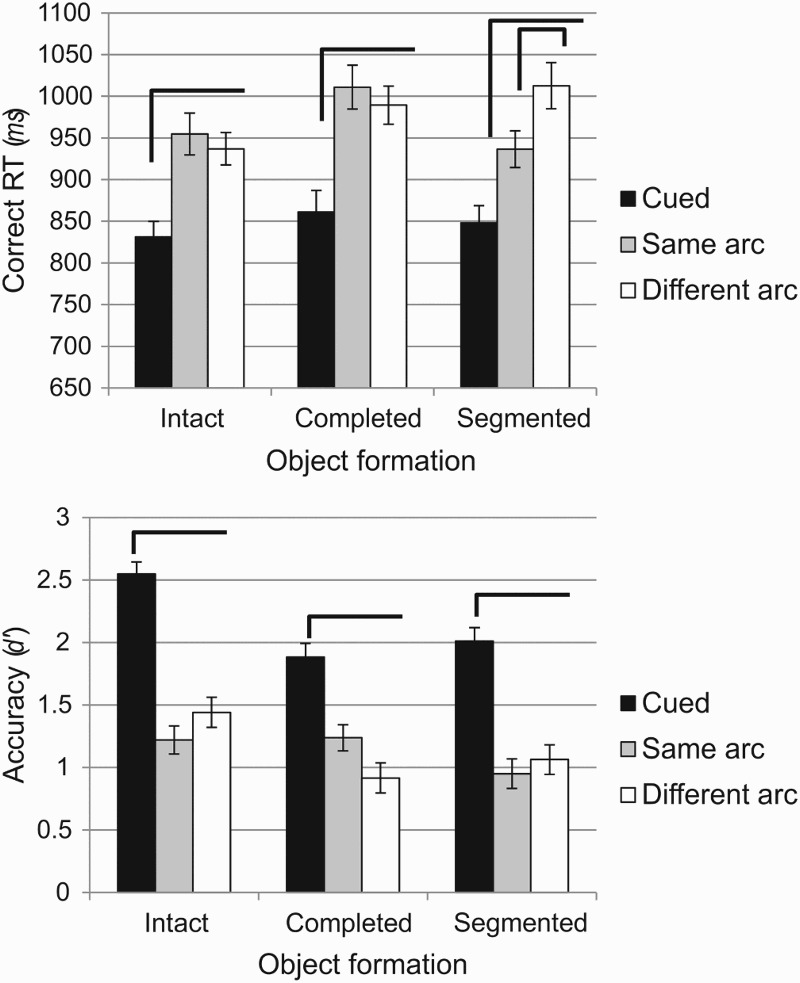
Performance as a function of probe location and object formation. Top panel: mean reaction time (ms) for correct responses; bottom panel: accuracy (d’). Brackets illustrate statistical differences within object formation at p < .05 (see text for details). Error bars represent SEM, corrected for between-subject variability (Cousineau, 2005).

Importantly, a different pattern of performance emerged as a function of which was the dependent variable in question. The contrast can be clearly observed on Figure 3, illustrating reaction time (top panel) and accuracy (bottom panel). Overall reaction time was not affected by changes in object formation, *F*(2, 50) = 1.224, *p* = .303, *MSE* = 33,951.45, 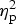 = .05, but it did vary as a function of probe location, *F*(1.51, 38.8) = 30, *p* < .001, *MSE* = 18,550.82, 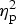 = .55. Most importantly, there was an interaction between probe location and object formation condition, *F*(4, 100) = 2.71, *p* = .034, *MSE* = 7817.01, 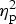 = .10. Planned comparisons at each level of object formation revealed that for the intact object condition, responses for cued probes were faster than for same and different arc probes (*p* < .001) while there was no difference between same and different arc probe locations (*p* > .99). Reaction times for the completed object condition followed the same pattern, with faster responses for cued probes than for same (*p* = .001) and different arc probes (*p* < .001), and no difference between the latter two (*p* > .99). Within the segmented condition, however, responding to cued probes was faster than responding to same (*p* = .001) and different (*p* < .001), but responses for same were also faster than responses for different arc probes (*p* = .018). Critically, therefore, even though they are physically segregated from the cued location, different arc probes are processed as readily as same arc probes if object-formation processes lead to them being on the same perceptual object as the cued location. On the other hand, if the physical segregation does not lead to object completion, then those different arc probes are disadvantaged relative to equidistant locations lying on the same intact object as the cued location.

With regards to accuracy, main effects of both object formation, *F*(2, 50) = 55.38, *p* < .001, *MSE* = 0.48, 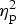 = .69, and probe location, *F*(2, 50) = 8.74, *p* = .001, *MSE* = 0.46, 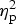 = .26, were significant, as was the interaction between them, *F*(4, 100) = 3.3, *p* = .014, *MSE* = 0.27, 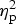 = .18). However, the critical interaction took a different form to that observed with the reaction time results. The pattern across probe locations was identical for the three object formation conditions, such that accuracy was superior for the cued probe (all *p*s < .001, except for cued compared to same arc probes within the completed object condition, where *p* = .005), and there were no differences between same and different arc probes in any object formation condition. So, unlike the case for reaction time, performance accuracy showed neither a same-object advantage nor an effect of perceptual organization of the display. Rather, the interaction here was due to higher accuracy for cued probes in the intact object condition than for those in the completed (*p* < .001) and segmented (*p* = .003) object conditions, and higher accuracy for different arc probes in the intact than for the completed (*p* = .03) condition. This pattern is unlikely to be due to a floor effect in performance for uncued locations given that *d*′ for those locations is consistently at or above 1. The overall superior performance in the intact condition, which appears to be the source of this interaction, may be due to the circular object being on top of the fan-shaped object, and hence closer (in terms of apparent depth) to the viewer, potentially granting it higher behavioural priority. In the completed condition, the apparent depth ordering is reversed, while for the segmented object it can be ambiguous. In any case, the observed effect is not critical for assessing the role of object-based mechanisms, so it is not discussed further.

In sum, the reaction time findings clearly indicate that change detection decisions were affected by the perceptual organization of the stimulus, such that information was more readily retrieved for target information located perceptually on the same object as the cued location. Critically, items within the same perceptual object as the cued feature were retrieved with the same speed, regardless of the presence of a physical discontinuity in the form of an occluding object. Therefore, the physical boundary did not in itself lead to a cost associated with the cued location being on a different object to the target. Rather, the perceptual organization of the scene determined the facility with which the probed information was processed in VSTM. Importantly, this effect was not evident for accuracy, as there was no statistical difference in performance between same and different arc probes within any of the object formation conditions.

### Discussion

Our aim here was to deliberately manipulate the way in which target information in a change-detection setting was organized into higher level objects—without changing the quantity or arrangement of target “items” per se—in order to discern how object formation may influence the way in which that target information is represented in VSTM. There are two key points to be made about the results; first, the fate of information in VSTM is a function of the higher order object formation processes to which that information is subject, and, second, not all measures of VSTM processing reveal this aspect of the representations in VSTM. So, in addition to the pronounced and general advantage for information at cued locations, processing of information located on the cued object showed greater facility than that for information equidistant from the cue, but lying on a different object. In addition, physical discontinuity between the cued and probed location per se was not sufficient to slow down performance as long as the object containing the locations was perceptually completed. This object-oriented pattern was not evident for accuracy, suggesting that the different measures are not equally sensitive to aspects of processing in VSTM. Just as object-level organization has been shown to play a key role in visual perception and attention (e.g., Chen, 2012; Egly, Driver, & Rafal, 1994; Reppa, Schmidt, & Leek, 2012), our results here show that such processes also need to be taken into account when investigating the representation of information in VSTM. This is especially so since such object formation processes occur in an obligatory fashion and therefore are likely to be affecting performance even in circumstances where they are not the focus of investigation.

Given the results here, there are a number of ways in which such factors may play out in methodologies examining the constraints on VSTM. The fact that a cue facilitates retrieval equivalently for equidistant features if they lie on the same object as the cue, but not if they lie on separate objects, means that changes in performance as a function of the number and type (e.g., with respect to shared/distinct features) of target “items” and their relation to the cue (e.g., spatial separation) may indicate effects of object formation on retrieval rather than of the nominal factor being manipulated. For example, since our results suggest equivalent activation of two features equidistant from a cue when those features belong to the same object, we might also expect output interference between such equivalently activated features to be greater than that under circumstances where the two features belong to separate objects and therefore are differentially activated by a cue from which they are equidistant. Such object-oriented mechanisms, therefore, may underpin effects such as spatial transposition gradients (e.g., Rerko, Oberauer, & Lin, 2014), which are ostensibly due to spatial separation, since increasing or decreasing the spatial separation between elements in a display will affect the likelihood that they form features of a single object (Elder & Goldberg, 2002). It is important to remember that while our methodology involved deliberate manipulation of object-level organization, there was no incentive on behalf of participants to encode the target information with respect to this organization. There was no predictive value in the cued location, with respect to either the object-level structure or the ensuing probe location. As such, it seems unlikely that cues to object formation would need to be deliberately processed in order to observe the sort of object-based effects reported here.

Similarly, manipulations involving the number of items and features will influence the precise way in which elements in the display are subject to object-formation processes, raising questions about the assumption that the number of objects or features introduced by the experimenter equals the number of objects encoded in VSTM (Luck & Vogel, 1997; Zhang & Luck, 2008). Presenting an array of six squares, for example, may result in the encoding of two or three perceptual objects by virtue of grouping on the basis of whatever cues are available in the display (e.g., proximity, similarity). As a result, there may be a mismatch in the inferred number of objects held in memory and the actual capacity (Brady & Tenenbaum, 2013). The issue of object formation may also be critical to interpretation of change detection results depending on the type of methodology used. For example, change detection is often assessed by presenting a stimulus array with multiple items and then at test using a single probe in isolation, or a single probe indicating the target for which a same/different decision needs to be made, accompanied by empty placeholders occupying the locations of the remaining nontarget items (Hardman & Cowan, 2015; Oberauer & Eichenberger, 2013; Rerko et al., 2014). The distinction between global, holistic—that is, in our terms, object level—processing and local, featural processing is well established (e.g., Navon, 1977; Wagemans et al., 2012) and has implications for a range of processes associated with visual processing (e.g., Torralba, Oliva, Castelhano, & Henderson, 2006; Wagemans et al., 2012). Therefore, by presenting a probe display that is a segment of what may have originally been encoded as an object-level representation, different patterns of performance will be expected compared to conditions where the whole display is re-presented and therefore affords object-level, rather than feature-level, matching (see e.g., Macken et al., 2015, for discussion of the analogous issue in auditory perception). Again, the influence of such a distinction would be expected to play out differently depending on the tendency for elements of the memory display to be integrated into higher level object representations.

Further general implication from the current findings is that clear evidence of the role of object-level representations in VSTM, where differential access to uncued probes was only observed when they were located on different perceptual objects to the cue, was evident for reaction times but not for accuracy data. Change detection accuracy did not vary based on whether the probe was in the same or in a different object relative to the cued feature. Benefits associated with the cued location are robustly established in the memory and attention literature (e.g., Schmidt, Vogel, Woodman, & Luck, 2002), and, as expected, both types of measurement here were complementary in replicating this effect. Schmidt et al. (2002), for example, compared directly the effect of predictive and nonpredictive cues on VSTM accuracy and found significant benefit for the cued item in both cases, albeit slightly larger when the cue was informative. Importantly, the conditions of the experiment very closely match the current one (same timing and number of stimuli, with the exception that perceptual organization was not explicitly manipulated). Woodman et al. (2003) also demonstrated persistent VSTM superiority for cued probes, even though the exogenous cue did not carry any strategic information. Therefore, exogenous cueing is very powerful and is known to produce pronounced benefits for VSTM. However, a continuous measure such as reaction time may be more sensitive to detecting aspects of processing of uncued probes than a categorical same/different judgement. In addition, participants were not time-limited when responding to the memory probe, which was visible until a response was made. Therefore, reaction times may more sensitively reveal the underlying structure of information in VSTM, reflected in the requirement for extended retrieval time in order to support a judgement when the probed information lies on an object other than the cued one.

A question worth considering is whether the pattern of performance observed here is the outcome of speed–accuracy trade-offs, since different arc reaction time is slower than same arc reaction time in the segmented condition, but also *d*′ for different arc is slightly higher than the same arc *d*′ value. Such a pattern may suggest a strategy that compensates for better accuracy by taking longer to make a decision. However, this is unlikely for a number of reasons. First, speed–accuracy trade-offs are typically dependent on the decision criterion starting point—that is, whether speed or accuracy is emphasized during task instructions—and also the probability of a target occurrence (Wagenmakers, Ratcliff, Gomez, & McKoon, 2008). In the current experiment, however, participants were instructed to balance speed and accuracy: One was not stressed more than the other. In addition, the probe location was not predicted by the cue, and each of the six possible targets was probed an equal amount of times. Participants were explicitly informed about this at the start of the experiment, so there was unlikely to be any bias in expectations. In addition, the reaction time difference between *different* and *same arc* probes for the *segmented* condition was 76 ms, while the equivalent *d*′ difference was merely 0.11 units, with largely overlapping error bars, suggesting an insignificant trade-off pattern. Of course, some fluctuation in response accuracy is expected, especially since the short exposure to the memory items (100 ms) makes the task rather challenging, and this fluctuation can be clearly seen in the pattern of *d*′ values within each object formation condition (Figure 3). However, the lack of statistical difference for the *d*′ measurement and the stability of the reaction time pattern suggest that even if there was an element of a trade-off, it is not the primary factor for the current result.

A further point worth considering is that presenting a cue prior to the study array may be considered as exerting an effect on the quality of perceptual input into VSTM, rather than internal VSTM processes per se. However, this possibility has been examined in detail by studies comparing pre- and postcues (i.e., cues presented during the retention interval between study and test), which found no difference between the two types—in terms of both accuracy benefits for items at the cued location (Griffin & Nobre, 2003; Schmidt et al., 2002), and object-based benefits for items perceptually grouped with it (Woodman et al., 2003). Considering also the suggested strong overlap between mechanisms responsible for perception, attention, and VSTM (Awh & Jonides, 2001; Theeuwes, Kramer, & Irwin, 2011), it does not appear likely that the object-based effects here are due to this particular aspect of our methodology.

Given all the issues discussed above, what the current results demonstrate is that the readiness with which information is retrieved from VSTM is affected by the object-level structure of the stimulus within which the target information occurs. It remains to be seen precisely how and when such typically overlooked object-level factors may be impacting on the outcome in studies of change detection that are focused on other stimulus properties as they may constrain VSTM. Importantly, such processes may form objects even from elements that are not physically continuous with, or even immediately adjacent to, each other. As such, just as it has become evident in studies of visual selection, studies of the structure and content of VSTM need to take object-level processing into account when quantifying performance under various conditions, and they cannot necessarily assume that the number and type of operational units in a display correspond to the contents of VSTM.
